# Whole-Exome Sequencing Identifies Homozygous *AFG3L2* Mutations in a Spastic Ataxia-Neuropathy Syndrome Linked to Mitochondrial *m*-AAA Proteases

**DOI:** 10.1371/journal.pgen.1002325

**Published:** 2011-10-13

**Authors:** Tyler Mark Pierson, David Adams, Florian Bonn, Paola Martinelli, Praveen F. Cherukuri, Jamie K. Teer, Nancy F. Hansen, Pedro Cruz, James C. Mullikin for the NISC Comparative Sequencing Program, Robert W. Blakesley, Gretchen Golas, Justin Kwan, Anthony Sandler, Karin Fuentes Fajardo, Thomas Markello, Cynthia Tifft, Craig Blackstone, Elena I. Rugarli, Thomas Langer, William A. Gahl, Camilo Toro

**Affiliations:** 1 NIH Undiagnosed Diseases Program, National Institutes of Health Office of Rare Diseases Research and National Human Genome Research Institute, Bethesda, Maryland, United States of America; 2 Neurogenetics Branch, National Institute of Neurological Disorders and Stroke, National Institutes of Health, Bethesda, Maryland, United States of America; 3 Office of the Clinical Director, National Human Genome Research Institute, National Institutes of Health, Bethesda, Maryland, United States of America; 4 Institute for Genetics, University of Cologne, Cologne, Germany; 5 Biocenter, University of Cologne, Cologne, Germany; 6 Genome Technology Branch, National Human Genome Research Institute, National Institutes of Health, Bethesda, Maryland, United States of America; 7 Genetic Disease Research Branch, National Human Genome Research Institute, National Institutes of Health, Bethesda Maryland, United States of America; 8 NIH Intramural Sequencing Center, National Human Genome Research Institute, National Institutes of Health, Bethesda, Maryland, United States of America; 9 EMG Section, National Institute of Neurological Disorders and Stroke, National Institutes of Health, Bethesda, Maryland, United States of America; 10 Division of Surgery, Children's National Medical Center, Washington, D.C., United States of America; 11 Institute for Genetics, Center for Molecular Medicine (CMMC), Cologne Excellence Cluster on Cellular Stress Responses in Aging-Associated Diseases (CECAD), University of Cologne, Cologne, Germany; 12 Max-Planck-Institute for Biology of Aging, Cologne, Germany; The Jackson Laboratory, United States of America

## Abstract

We report an early onset spastic ataxia-neuropathy syndrome in two brothers of a consanguineous family characterized clinically by lower extremity spasticity, peripheral neuropathy, ptosis, oculomotor apraxia, dystonia, cerebellar atrophy, and progressive myoclonic epilepsy. Whole-exome sequencing identified a homozygous missense mutation (c.1847G>A; p.Y616C) in *AFG3L2*, encoding a subunit of an *m*-AAA protease. *m*-AAA proteases reside in the mitochondrial inner membrane and are responsible for removal of damaged or misfolded proteins and proteolytic activation of essential mitochondrial proteins. AFG3L2 forms either a homo-oligomeric isoenzyme or a hetero-oligomeric complex with paraplegin, a homologous protein mutated in hereditary spastic paraplegia type 7 (SPG7). Heterozygous loss-of-function mutations in *AFG3L2* cause autosomal-dominant spinocerebellar ataxia type 28 (SCA28), a disorder whose phenotype is strikingly different from that of our patients. As defined in yeast complementation assays, the AFG3L2^Y616C^ gene product is a hypomorphic variant that exhibited oligomerization defects in yeast as well as in patient fibroblasts. Specifically, the formation of AFG3L2^Y616C^ complexes was impaired, both with itself and to a greater extent with paraplegin. This produced an early-onset clinical syndrome that combines the severe phenotypes of SPG7 and SCA28, in additional to other “mitochondrial” features such as oculomotor apraxia, extrapyramidal dysfunction, and myoclonic epilepsy. These findings expand the phenotype associated with *AFG3L2* mutations and suggest that *AFG3L2-*related disease should be considered in the differential diagnosis of spastic ataxias.

## Author Summary

Mitochondria are cellular organelles important for converting sugar or fats into energy that cells can use for their functions and survival. Many neurological diseases are the result of mitochondrial dysfunction as affected cells are unable to cope with lowered energy supplies and increased oxidative stress. These deficiencies cause accumulation of cellular damage and eventually cell death. Spastic ataxias are neurological disorders involving cells with large energy requirements, the cerebellar Purkinje cells and the cerebral upper motor neurons. When these cells function improperly or die, individuals develop symptoms of incoordination (ataxia) and abnormal muscle tone in their legs (spastic paraplegia). Using emerging techniques of whole-exome sequencing we discovered that homozygous mutations in the *AFG3L2* gene caused spastic ataxia in two brothers of a consanguineous family. *AFG3L2* encodes a subunit of mitochondrial matrix proteases (*m*-AAA proteases) that regulate the functional integrity of mitochondria. Heterozygous mutations in *AFG3L2* were previously found to cause a disorder involving the Purkinje cells of the cerebellum resulting in ataxia. Interestingly, another isoform of *m*-AAA proteases consists of AFG3L2 complexing with paraplegin, a similar protein associated with a hereditary spastic paraplegia. Our analysis provides insight into why different mutations in *m*-AAA protease subunits cause different neurological disorders.

## Introduction

*AFG3L2* encodes a subunit of the *m*-AAA class of mitochondrial proteases [1], [2]. These ATP-dependent metallopeptidases assemble into large proteolytic complexes in the inner membrane of mitochondria and function to ensure mitochondrial protein quality control through the degradation of misfolded proteins and the maturation of essential proteins [3]. In humans, *m*-AAA proteases assemble into different isoenzymes: homo-oligomeric complexes of AFG3L2 subunits or hetero-oligomeric complexes of AFG3L2 with paraplegin (encoded by *SPG7*). Notably, paraplegin is incapable of self-assembling into homo-oligomers and requires AFG3L2 for function. Mutations in *AFG3L2* cause autosomal dominant spinocerebellar ataxia 28 (SCA28; MIM #610246), a recently recognized and rare disorder characterized clinically by adult-onset dysarthria, ptosis and cerebellar ataxia [1], [4], [5]. Mutations in *SPG7*, on the other hand, are associated with another type of *m*-AAA-associated neurological disease, autosomal recessive hereditary spastic paraplegia type 7 (SPG7; MIM #607259). This disorder is characterized by adult-onset spasticity and weakness of the lower extremities due to a length-dependent axonopathy of corticospinal motor neurons [6], [7]. The phenotypic variation between these two disorders is particularly remarkable since these proteins are *m*-AAA protease subunits that interact with one another.

Transgenic mouse models have been informative in characterizing the functional role of *m*-AAA complexes in human disease. While *Spg7* ^+/-^ mice are normal, *Spg7* ^-/-^ null mice accurately phenocopy human SPG7 with a late-onset impairment of motor performance and degeneration of long spinal and peripheral axons [8]. *Afg3l2* ^-/-^ null mice, which have no human counterpart to date, exhibit a severe neurodegenerative phenotype associated with deficient axonal radial growth and delayed myelination, resulting in poor central and peripheral axonal development [1], [9]. These mice develop hind limb paresis by P7, which progresses to complete paralysis and death by P16 [1]. Predictably, *Afg3l2* ^+/-^ mice have late-onset progressive motor incoordination and Purkinje cell degeneration similar to human SCA28 [1], [9]. Of particular interest, double mutant *Spg7^-/-^Afg3l2^+/-^* mice exhibit a unique phenotype with severe early-onset spasticity and impairment of cerebellar function that is associated with axonal and cerebellar degeneration. This latter result reflects the accelerated progression of each phenotype and indicates that decreased dosage of both proteins could have a synergistic effect on the expression of these respective disorders [10]. Similar alterations in the dosage of *m*-AAA activity in humans would be predicted to have comparable phenotypic findings.

We now describe two teenage brothers, of a consanguineous family, with a novel homozygous missense mutation in *AFG3L2* initially identified by whole exome sequencing (WES). Yeast studies demonstrate that this mutation decreases cellular *m*-AAA activity due to impaired oligomerization with itself as well as with paraplegin. As a result, our patients present with an early-onset phenotype combining features of SPG7 and SCA28 resembling the accelerated phenotype seen in compound *Spg7^-/-^Afg3l2^+/-^* mice. Furthermore, the brothers have the additional findings of oculomotor apraxia, dystonia, and progressive myoclonic epilepsy, which are common features in mitochondrial disorders and likely result from reduced *m*-AAA activity in other regions of the CNS. Our findings expand the spectrum of neurological features associated with defective *m*-AAA protease activity, and highlight the phenotypic variation of *m*-AAA disorders as a result of the type of mutation and subunit involved.

## Results

### Patient examination and genetic evaluation

**Figure 1:**
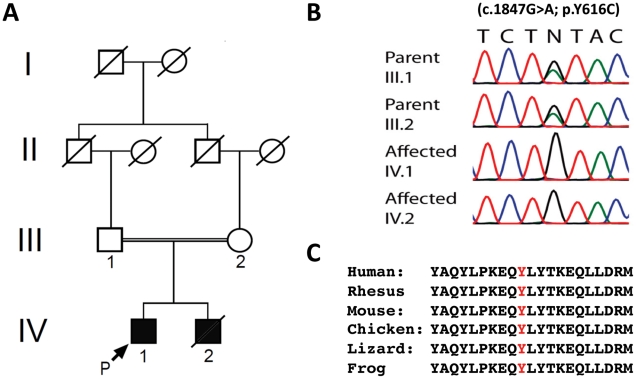
Genetic analysis of family with early onset spastic ataxia-neuropathy syndrome. (A) Pedigree of family. (B) Sequencing of the c.1847G>A; p.Y616C *AFG3L2* mutation in family members. (C) Alignment of amino acid sequences from AFG3L2 of several vertebrate species indicates that Y616 and the flanking residues are highly conserved.

The parents of the two affected brothers were first cousins of Hispanic origin with no family history for neurodegenerative disease (Figure 1A). The siblings were born thirteen months apart in Colombia without complications. Their disease courses were similar, with the younger sibling's being more severe. The older brother (IV-1) had normal development until exhibiting a spastic gait in his second year. The younger brother (IV-2) never ambulated independently. At eight years of age, each developed progressive myoclonic epilepsy with stimulus-induced myoclonus associated with generalized tonic-clonic and myoclonic seizures. This was followed by progressive dysarthria, dysphagia, and motor degeneration, with the older sibling eventually losing his ability to ambulate. Both subsequently developed lower extremity weakness and distal muscle atrophy.

IV-2 died at 13 years of age from pneumonia-related complications, and our evaluation was limited to other physician's notes and genetic evaluation. IV-1's examination revealed spastic paraparesis in the lower extremities as well as appendicular dysmetria, dysdiadochokinesia, and ataxic dysarthria. He also had decreased muscle bulk and strength in his lower extremities. Additional findings included ptosis, oculomotor apraxia, dystonic movements, and stimulus-induced myoclonus. Fundoscopic and ophthalmological exams were unremarkable. His cognition was normal. In addition, both parents were without neurological complaints and had normal neurological and ophthalmological exams.

**Figure 2:**
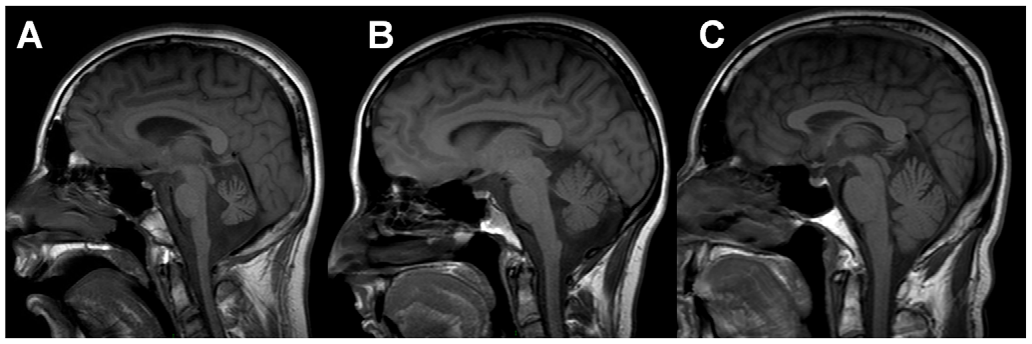
Neuroimaging of family members. T1 sagittal magnetic resonance imaging of the brain of IV-2 (A) reveals notable cerebellar atrophy. His asymptomatic mother, III-2 (B), has mild cerebellar atrophy, while his asymptomatic father, III-1 (C) has normal imaging.

Diagnostic testing of IV-1 revealed multiple abnormalities (Table 1). Brain MRI without contrast revealed moderate cerebellar atrophy (Figure 2A). Nerve conduction studies showed an axonal sensorimotor neuropathy affecting his lower extremities, and sural nerve biopsy revealed mild “onion bulbing” suggestive of a mild chronic demyelinating/remyelinating process. Muscle biopsy histology by light microscopy was within normal limits and without ragged red fibers; however, transmission electron microscopy revealed misplaced mitochondria associated with large lipid droplets. Muscle samples also had decreased mtDNA copy number. Finally, an electroencephalogram was consistent with progressive myoclonic epilepsy (Table 1). His parents underwent a less extensive evaluation. Parental nerve conduction studies were normal. Brain MRIs revealed that the father (III-1; 58 years old) was within normal limits; however, the mother (III-2; 39 years old) had mild cerebellar atrophy (Figure 2B).

**Table 1:** Clinical and Laboratory Data for Patient IV-1.

Diagnostic Evaluation	Results
Brain MRI	Moderate cerebellar/pontine atrophy, mild thinning of corpus callosum otherwise the supratentorial brain was unremarkable.
Brain MR Spectroscopy	Low NAA values in pons and cerebellum.
Nerve conduction studies	Axonal sensorimotor peripheral neuropathy in bilateral lower extremities; upper extremity testing was normal.
Electromyography(left tibialis anterior)	Membrane irritability, representing active denervation, and polyphasic motor units representing denervation/reinnervation
Sural nerve biopsy	Chronic demyelination/remylination process represented by mild onion bulbing (Schwann cell processes around a central axon). There was also focal small axonal sprouting suggestive of axonal regeneration.
Skeletal muscle biopsy	Relatively normal structure and biochemical staining. Transmission electron microscopy evaluation revealed an increased number of lipid droplets between the myofibrils and sarcolemmal region that were often associated with mitochondria. Mitochondria showed variation in shape and size including elongated morphology.
Skeletal muscle mtDNA copy number	2161 (normal 2951-4427)
Electroencephalogram	Diffuse slowing/disorganization, fronto-central spike/waves, and myoclonic stimulus-induced seizures during photic stimulation.

Since prior genetic testing had not yielded a diagnosis (see Materials and Methods), we performed WES to search for pathogenic DNA variants [11], [12]. Individual nucleotide variants were evaluated and filtered using several methods including their presence or absence in dbSNP, segregation analysis, and the estimation of pathogenic potential using amino-acid conservation programs (see Materials and Methods and Tables S1, S2, S3)[13]. An average of 8585 missense and 87 nonsense changes were identified in each family member tested. The presence of consanguinity and the fact that the parents appeared largely asymptomatic suggested that the patients would likely be homozygous for any disease-causing mutations, and therefore a homozygous autosomal-recessive model was applied. Final analysis yielded two variant-containing candidate genes that fulfilled our selection criteria (Table S3). One candidate gene, *DMGDH*, encoding dimethylglycine dehydrogenase (MIM #605850), was previously associated with a syndrome of elevated serum creatine kinase and an unusual fish-like body odor [14], [15]. This was not consistent with the siblings' phenotype. The other candidate gene, *AFG3L2*, had recently been associated with autosomal-dominant SCA28 [2], [4], [5]. Because the brothers had the features of a cerebellar ataxia, we considered the homozygous *AFG3L2* mutation to be the more likely cause of the siblings' disorder. This variant consisted of a c.1847A>G mutation in exon 15, which resulted in the missense change, p.Y616C (Figure 1B). This tyrosine residue is located at the beginning of the proteolytic domain and is highly conserved among vertebrates, including mouse, chicken, frog, lizard, and stickleback (Figure 1C)[16].

### AFG3L2^Y616C^ is functionally impaired, but retains ATPase and proteolytic activities

Previous studies have shown that yeast is a useful model system to assess the functional activity of human *m*-AAA proteases and to investigate in detail the pathogenic mechanisms of mutations in *m*-AAA protease subunits [17]. *m*-AAA proteases are evolutionarily conserved, with *S. cerevisiae* possessing only hetero-oligomeric *m*-AAA protease complexes composed of Yta10 and Yta12 subunits. These proteins can be substituted with the human orthologues in order to study their respective chemistries. Yeast cells lacking Yta10 and Yta12 (Δ*yta10*Δ*yta12* cells) are incapable of proteolytically processing the nuclear-encoded mitochondrial protein MrpL32, a component of mitochondrial ribosomes. Impaired ribosome assembly in the absence of the *m*-AAA protease abolishes the synthesis of mitochondrial-encoded respiratory chain subunits and impairs aerobic respiration [18]. Thus, the activity of *m*-AAA proteases in yeast cells can be monitored either directly, by western blot analysis of the conversion of MrpL32 from its precursor to its mature form, or indirectly, by assessing respiratory growth on non-fermentable carbon sources such as glycerol [17], [19].

**Figure 3:**
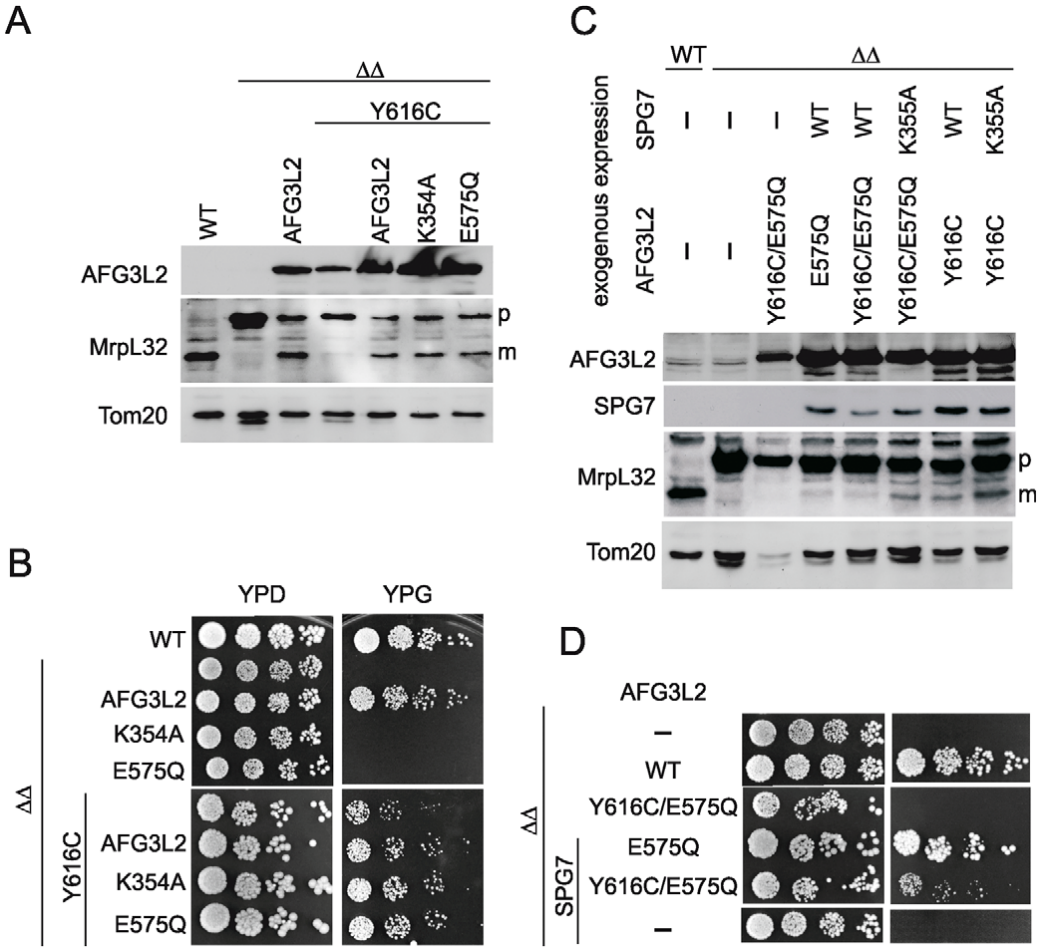
MrpL32 maturation and yeast complementation assays for the evaluation of AFG3L2^Y616C^ activity. (A) Protein expression of AFG3L2 in Δ*yta10*Δ*yta12* cells was analyzed by SDS-PAGE and immunoblotting using AFG3L2-specific antibodies. Maturation of MrpL32, a substrate of *m*-AAA proteases, was monitored in isolated mitochondria by immunoblotting using polyclonal antisera directed against MrpL32. The outer membrane protein Tom20 was used as a loading control. (B) Respiratory growth of Δ*yta10*Δ*yta12* cells expressing human *m*-AAA protease subunits. To assess the functional activity of homo-oligomeric *m*-AAA proteases, AFG3L2^Y616C^ was expressed in Δ*yta10*Δ*yta12* cells alone or co-expressed with AFG3L2, AFG3L2^E575Q^ (proteolytic site mutant) or AFG3L2^K354A^ (ATPase domain-Walker A motif mutant) where indicated. Cell growth was analyzed at 30°C on glucose- (YPD) or glycerol containing (YPG) media. (C) Protein expression and maturation of MrpL32 were monitored in Δ*yta10*Δ*yta12* cells expressing the indicated variants of human *m*-AAA protease subunits as in (A). (D) To monitor the activity of hetero-oligomeric *m*-AAA complexes, AFG3L2^Y616C^ and AFG3L2^Y616C/E575Q^ were expressed with paraplegin (SPG7) or SPG7^K355A^ (Walker A motif mutant). Cell growth was analyzed at 30°C on glucose- (YPD) or glycerol-containing (YPG) media.

*m*-AAA proteases form hexameric complexes [20]. Two isoforms can be distinguished in human mitochondria: a homo-oligomeric form comprised of AFG3L2 subunits and a hetero-oligomeric form containing AFG3L2 and paraplegin subunits in equimolar ratios. We examined the functional activity of AFG3L2^Y616C^ in the context of both isoenzymes. Expression of only human AFG3L2 in Δ*yta10*Δ*yta12* cells restores maturation of MrpL32 and respiratory growth on glycerol-containing media, demonstrating functional conservation of yeast and homo-oligomeric human *m*-AAA proteases (Figure 3A, 3B) [17], [19]. Remarkably, expression of AFG3L2^Y616C^ impaired processing of MrpL32 in comparison to cells harboring AFG3L2 (Figure 3A). However, maturation was not completely inhibited, which allowed for some respiratory growth of Δ*yta10*Δ*yta12* cells (Figure 3B). We conclude that the Y616 mutation of AFG3L2 interferes with, but does not completely inhibit, the activity of the homo-oligomeric *m*-AAA protease.

Respiratory growth of yeast cells and MrpL32 processing depend on both ATPase and proteolytic activities of the *m*-AAA protease. Mutations of the Walker A site of AFG3L2's AAA domain (K354A) or within its proteolytic center (E575Q) inactivated the homo-oligomeric protease and abolished respiratory growth (Figure 3B). In order to directly monitor both activities of the AFG3L2^Y616C^ variant, we exploited our previous observation that functional deficiencies in individual domains of *m*-AAA protease subunits can be complemented by the presence of wild-type domains in other subunits of the assembled *m*-AAA ring complexes [21]. In other words, assembled *m*-AAA protease complexes composed of wild-type and inactive mutant subunits are functionally active [21]. Co-expression of AFG3L2 with AFG3L2^Y616C^ in Δ*yta10*Δ*yta12* cells substantially increased MrpL32 processing and respiratory growth when compared to cells expressing only AFG3L2^Y616C^ (Figure 3A, 3B). MrpL32 processing and respiratory growth of Δ*yta10*Δ*yta12* cells were restored to a similar extent upon co-expression of AFG3L2^Y616C^ with either of the two inactive AFG3L2 variants (AFG3L2^K354A^ or AFG3L2^E575Q^) (Figure 3A, 3B). As the AFG3L2^K354A^ and AFG3L2^E575Q^ subunits lack ATPase and proteolytic activity, respectively, and are functionally inactive as homo-oligomeric complexes, the significantly improved MrpL32 processing and respiratory growth upon co-expression with AFG3L2^Y616C^ indicates intersubunit complementation of both ATPase and proteolytic activities [21].

These experiments demonstrate that homo-oligomeric *m*-AAA protease complexes composed of AFG3L2^Y616C^ subunits are functionally impaired, but retain both ATPase and proteolytic activities. We conclude that AFG3L2^Y616C^ is not a loss-of-function mutant, but appears to be hypomorphic. This is in striking contrast to AFG3L2 mutations causing SCA28 that fail to process MrpL32 and do not promote respiratory growth in Δ*yta10*Δ*yta12* cells [2].

### AFG3L2^Y616C^ affects the activity of hetero-oligomeric m-AAA proteases containing paraplegin

To examine the functional activity of AFG3L2^Y616C^ in the context of the hetero-oligomeric *m*-AAA isoenzyme, we co-expressed AFG3L2^Y616C^ with paraplegin or an inactive variant harboring a mutation in the ATPase domain (SPG7^K355A^) in Δ*yta10*Δ*yta12* cells. We observed MrpL32 processing (Figure 3C) and respiratory growth (Figure 3D), indicating the presence of functionally active *m*-AAA protease in these cells. The interpretation of these experiments, however, is complex, since AFG3L2^Y616C^ may self-assemble and/or co-assemble with paraplegin. Thus, it cannot be distinguished whether respiratory growth is maintained by homo-oligomeric AFG3L2^Y616C^ complexes or by hetero-oligomeric *m*-AAA proteases composed of AFG3L2^Y616C^ and paraplegin.

We therefore introduced a point mutation in the proteolytic domains of AFG3L2 (AFG3L2^E575Q^) and AFG3L2^Y616C^ (AFG3L2^Y616C/E575Q^). This mutation renders each variant of AFG3L2 proteolytically inactive and abolishes their ability to restore respiratory growth of Δ*yta10*Δ*yta12* cells (Figure 3D) [17], [19]. However, when AFG3L2^E575Q^ and paraplegin were co-expressed in Δ*yta10*Δ*yta12* cells, respiratory growth was restored (Figure 3D) [17], [19]. This is due to paraplegin, which is unable to self-assemble, forming hetero-oligomeric complexes with AFG3L2^E575Q^; in this context, paraplegin provides the proteolytic activity within the *m*-AAA protease ring complexes through intersubunit complementation [19], [21]. In stark contrast to these results with AFG3L2^E575Q^, AFG3L2^Y616C/E575Q^ did not allow respiratory growth when co-expressed with paraplegin (Figure 3B), indicating that the activity of the AFG3L2^Y616C^ variant is impaired in the context of a hetero-oligomeric *m*-AAA isoenzyme with paraplegin.

### Impaired assembly of AFG3L2^Y616C^ into m-AAA protease complexes

**Figure 4:**
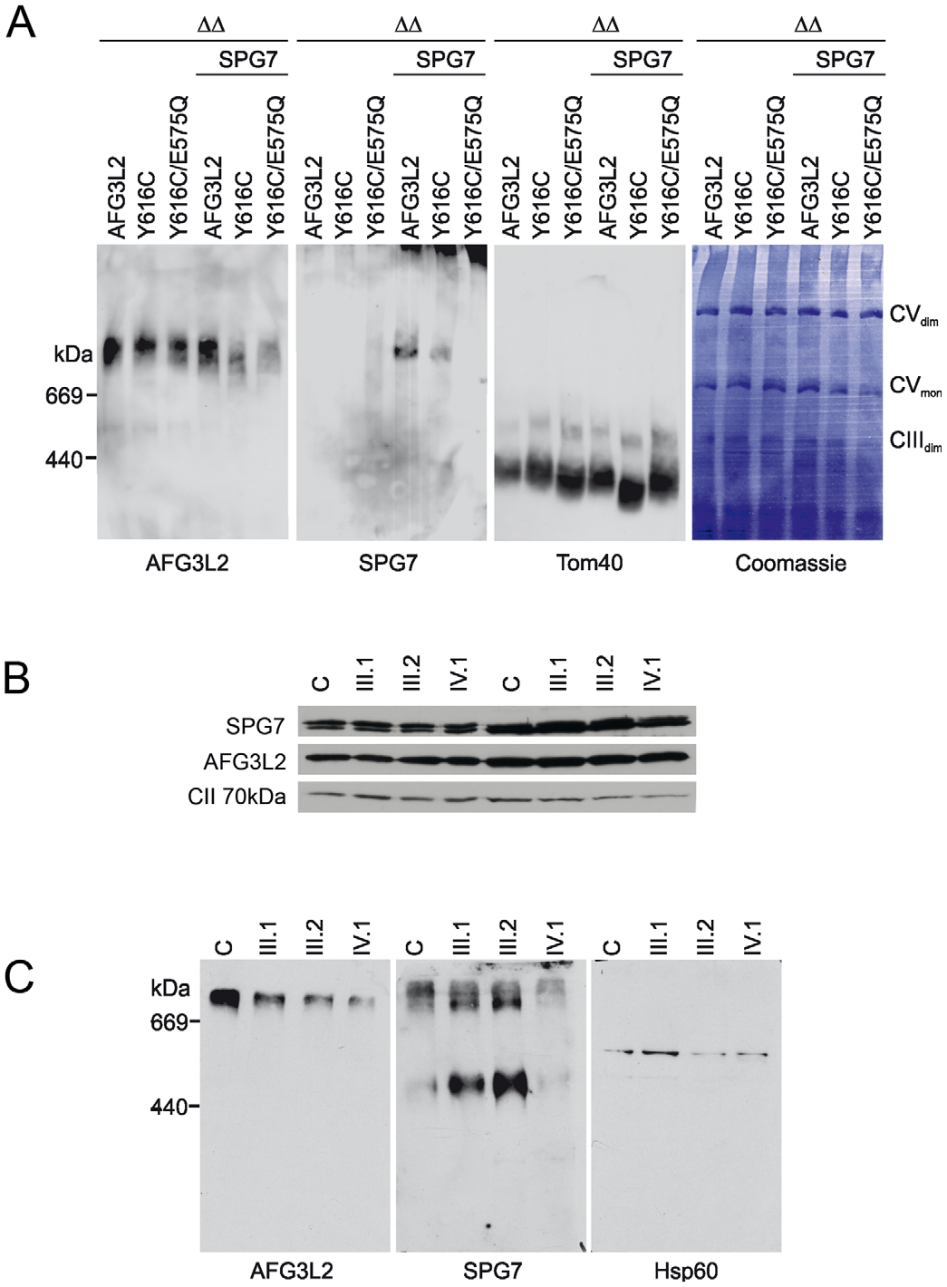
Assembly of AFG3L2, AFG3L2^Y616C^, and AFG3L2^Y616C/E575Q^ in mitochondria. (A) Mitochondrial extracts (100 µg protein) harbouring AFG3L2 (and variants thereof) and paraplegin (SPG7) as indicated were isolated from Δ*yta10*Δ*yta12* cells and solubilized with digitonin (1% (w/v)) at a concentration of 5 mg/ml. Extracts were analyzed by BN-PAGE, transferred onto a PVDF membrane and stained with Coomassie blue G-250 (right panel). After destaining, the membrane was used for immunoblotting using AFG3L2- or SPG7-specific antibodies. The outer membrane protein Tom40 was used as a loading control. Thyroglobulin (669 kDa) and apoferritin (440 kDa) were used for size calibration. (B) Mitoplasts were prepared from primary fibroblasts of family members and solubilized with digitonin. Soluble and pellet fractions were analysed by SDS-PAGE using AFG3L2- and paraplegin-specific antibodies. No alteration of the steady-state levels of the two proteins was observed in the proband (IV.1). (C) To detect assembled *m*-AAA proteases in the patient cell line, the same preparations were analyzed by BN-PAGE using AFG3L2- or paraplegin-specific antibodies. HSP60 was used for calibration. WT: wild-type; ΔΔ: Δ*yta10*Δ*yta12*; CVmon: complex V monomer; CVdim: complex V dimer; CIIIdim: complex III dimer.

Amino acid Y616 is located in close proximity to the protomer interface in structural models of hexameric *m*-AAA protease ring complexes [2], [20]. Interestingly, amino acid residues in close proximity to this region are involved in oligomerization of yeast *m*-AAA proteases [20]. We therefore assessed the assembly of AFG3L2^Y616C^ with itself or with paraplegin in Δ*yta10*Δ*yta12* cells. Mitochondrial extracts were isolated from Δ*yta10*Δ*yta12* cells expressing different AFG3L2 variants alone or in combination with paraplegin and analyzed by blue native-polyacrylamide gel electrophoresis (BN-PAGE)(Figure 4A). When compared to AFG3L2, the assembly of AFG3L2^Y616C^ with paraplegin was severely inhibited, while the formation of AFG3L2^Y616C^ homo-oligomers was impaired to a consistently lesser degree (Figure 4A).

To further substantiate these results, mitoplasts (isolated mitochondria without their outer membranes) were prepared from primary skin fibroblasts from family members and used to test the stability of AFG3L2^Y616C^, as well as its assembly into homo-oligomeric or hetero-oligomeric *m*-AAA complexes. AFG3L2^Y616C^ and paraplegin are present at normal levels in patient fibroblasts both in the soluble and pellet fractions, demonstrating that the mutation does not affect the solubility or the stability of the AFG3L2 protein (Figure 4B). However, BN-PAGE analysis of the same mitoplast preparations showed a reduced amount of assembled AFG3L2-containing complexes in fibroblasts of patient IV-1; moreover, hetero-oligomeric complexes harboring paraplegin were drastically reduced (Figure 4C). Consistent with these findings, the heterozygous parents displayed intermediate levels of assembled complexes (Figure 4C). Furthermore, these results are not due to abnormal trafficking of AFG3L2^Y616C^, as this variant's targeting to mitochondria was not impaired when overexpressed in cells (Figure S1).

Taken together, these experiments provide evidence that the primary pathogenic process of AFG3L2^Y616C^ involves impaired oligomerization with itself and paraplegin, leading to remarkably low levels of functionally active *m*-AAA protease complexes.

## Discussion

This study links an early onset spastic-ataxia-neuropathy syndrome to a unique homozygous mutation in *AFG3L2* in the absence of any associated *SPG7* mutations. Studies in yeast and patient fibroblasts reveal that the AFG3L2^Y616C^ mutant is a hypomorphic variant with reduced respiratory capability. This reduction is a consequence of its impaired ability to undergo oligomerization with itself or, in an even more pronounced manner, with paraplegin. Recent experiments with yeast *m*-AAA orthologues indicate a crucial role of the proteolytic domains for protease assembly. Interestingly, within a structural model of *m*-AAA proteases, the Y616 residue is in close proximity to amino acids that determine hetero-oligomerization of the yeast orthologue and prevent its homo-oligomerization [20]. Nevertheless, further interpretations regarding why the Y616C mutation has its specific effects on AFG3L2 and paraplegin oligomerization are difficult to make without the availability of crystal structures for both homo- and hetero-oligomeric complexes. In the homozygous context of our patients, the reduced levels of homo-oligomeric *m*-AAA proteases and the almost complete absence of hetero-oligomeric *m*-AAA isoenzymes explain the striking combination of clinical features of both SPG7 and SCA28, including spastic paraplegia, ptosis, and cerebellar ataxia [2], [4], [6], [22].

These features could have been anticipated by the phenotype of transgenic *Afg3l2* ^+/-^ *Spg7* ^-/-^ mice, whose similarly reduced dosage of m-AAA activity produced a comparable syndrome. Our genetic and biochemical findings were consistent with these transgenic mouse models of *m*-AAA dysfunction and indicate a critical role for the dosage of *m*-AAA protease activity in the maintenance of neuronal functions. Homo- and hetero-oligomeric *m*-AAA isoenzymes exhibit overlapping activities and can partially substitute for each other [19]; however as seen in *Afg3l2* ^+/-^ *Spg7* ^-/-^ mice, the combination of absent paraplegin and deficient AFG3L2 creates a severe phenotype. Our patients' features closely resemble those of the *Afg3l2* ^+/-^ *Spg7* ^-/-^ mice -- early-onset axonopathy and cerebellar degeneration, as well as mitochondrial DNA depletion [10]. AFG3L2^Y616C^, whose assembly with paraplegin is impaired, may lead to an effective loss of paraplegin function in the patients as paraplegin is unable to self-assemble and requires oligomerization with AFG3L2 for function. Mutations in this region of AFG3L2 may create a similar combined effect, as seen in *Afg3l2* ^+/-^ *Spg7* ^-/-^ mice, of *Spg7* deletion and *Afg3l2* heterozygosity, i.e., the loss of hetero-oligomeric *m*-AAA proteases containing paraplegin combined with reduced levels of functional AFG3L2. The end result would be decreased total *m*-AAA activity in affected neural cells producing a combined SCA28/SPG7 phenotype.

Our patients also exhibit additional neurological findings seen in other mitochondrial disorders, including oculomotor apraxia, dystonia, and progressive myotonic epilepsy [23]. The presence of these mitochondrial symptoms is consistent with the known function of AFG3L2 and paraplegin in mitochondrial respiration. Specifically, these proteins' involvement in mitochondrial quality control, i.e., the removal of misfolded or damaged mitochondrial proteins and activation of proteins that are essential for aerobic respiration [3]. Dystonia was previously observed in a family whose affected members carried an 18p chromosomal deletion that included *AFG3L2* [24], and *Afg3l2* ^+/-^ *Spg7* ^-/-^ mice also exhibited dystonic features [10]. The absence of our patients' other additional mitochondrial symptoms in *Afg3l2* ^+/-^ *Spg7* ^-/-^ mice means these mice were not an exact phenocopy; however, this may be due to the compensatory effects of an additional murine *m*-AAA protease subunit, Afg3l1, which has overlapping activities with Afg3l2 [19], [25]. Overall, it is likely that decreased *m*-AAA dosage in regions of the CNS usually unaffected by SCA28 or SPG7 are the cause of these features, but in a consanguineous family it is important to remember other genes may be modifying the phenotype.

Yeast complementation studies help explain the phenotypes of all the members of our patients' family. In the proband's cells, assembled *m*-AAA proteases were present at decreased levels. In this respect, the *AFG3L2^Y616C^* mutation behaved much differently as compared to the previously described loss-of-function *AFG3L2* variants associated with dominant SCA28 [2]. The decreased, but present, functional activity of AFG3L2^Y616C^ likely explains the lack of an obvious clinical phenotype in the heterozygous parents of the affected patients. In their cells, the combined expression of both the AFG3L2^Y616C^ and wild-type alleles appears to provide a sufficient amount of *m*-AAA protease activity to overcome a theoretical threshold for cerebellar disease. That threshold is likely greater than 50% of normal activity, because most heterozygotes for loss-of-function *AFG3L2* mutations are symptomatic. However, it should be recognized that previously reported SCA28 patients had late-onset cerebellar ataxia and, although the brain MRI of our proband's father was within normal limits, the mother had asymptomatic, mild cerebellar atrophy. This result indicates that it remains to be seen whether any family member heterozygous for *AFG3L2^Y616C^* will develop neurological dysfunction later in life.

Analysis of the AFG3L2^Y616C^ mutation identified here, together with data from transgenic mice and patients with either SCA28 or SPG7, link a spectrum of neurological phenotypes to specific mutations in *m*-AAA protease subunits. This spectrum includes: homozygous loss-of-function mutations in *SPG7* resulting in the absence of hetero-oligomeric *m*-AAA isoenzymes (associated with SPG7); heterozygous loss-of-function mutations in *AFG3L2* decreasing the dosage of both homo- and hetero-oligomeric forms of the *m*-AAA proteases (associated with SCA28); and homozygous AFG3L2^Y616C^ mutations also affecting both isoforms and further reducing the residual cellular *m*-AAA protease activity (associated with the early-onset spastic ataxia-neuropathy syndrome described here). Other genetic combinations of defective subunits could also cause neurological dysfunction, with decreased dosage of *m*-AAA activity resulting from various mixtures of heterozygous or homozygous mutations in *AFG3L2* and/or *SPG7*. The inheritance could appear to be either autosomal recessive and/or dominant indicating that genetic testing of *AFG3L2* and *SPG7* in any individuals with spastic ataxia, whether or not mitochondrial symptoms are present, may identify additional patients with *m-*AAA-related neurological disease.

Although the presently described syndrome shares features with SCA28 and prominent spasticity clearly extends the phenotypic spectrum associated with *AFG3L2* mutations, it appears different from SCA28 in several ways, specifically: 1) much earlier onset of symptoms, 2) spastic paraplegia as the earliest and predominant feature, and 3) presence of a peripheral neuropathy. As SCA28 is also identified with autosomal dominant inheritance, we feel that the current syndrome is distinct and should be classified separately as an AFG3L2-associated spastic-ataxia-neuropathy syndrome. With the identification of other patients with similar phenotypes we may be able to delineate the consistent and variable features of AFG3L2-related disorders and whether other mutations in *AFG3L2* and *SPG7* will only cause a spastic-ataxia-neuropathy syndrome or consistently produce an expanded “PME-spastic-ataxia-neuropathy” syndrome.

## Materials and Methods

### Ethics statement

Clinical and laboratory studies were approved by the NHGRI IRB. Patients and family members who were enrolled in the clinical protocol gave written informed consent, specifically including WES.

### Relevant previous genetic and metabolic workup

Sanger sequencing of the following genes was within normal limits: *SPG7*, *CLN8*, *COX10*, *DLD*, *EPM1*, *EPM2A*, *EPM2B*, *PANK2*, *PLA2G6*, *PRICKLE1*, *SCO1*, *SCO2*, *SURF1*, as was an analysis of mtDNA for point mutations, deletions and duplications. A lysosomal enzyme panel and buffy coat electron microscopy were also normal.

### DNA samples

Genomic DNA was extracted from peripheral whole blood, using the Gentra Puregene Blood kit (Qiagen) per manufacturer's standards. An additional chloroform-phenol extraction step was carried out to neutralize infectious agents.

### Genotyping

Illumina HumanOmni1-Quad genotyping arrays were run for one affected child and parents. The deceased brother's DNA was not analyzed on the SNP chip due to sample limitations. Analyses including homozygosity mapping were carried out using Illumina GenomeStudio Software. SNP chip data were also used to verify exome sample IDs for quality control purposes, and to calculate sensitivity and specificity for genotype calling in exome sequence data.

### Next-generation sequencing and variant analysis

Solution hybridization exome capture was carried out using the Sureselect Human All exon System (Agilent Technologies, Santa Clara, CA). This technique uses biotinylated RNA baits to hybridize to sequences that correspond to exons [26]. Manufacturer's protocol version 1.0 compatible with Illumina paired-end sequencing was used, with the exception that DNA fragment size and quality was measured using a 2% agarose gel stained with Sybr Gold instead of using an Agilent Bioanalyser. The manufacturer's specifications state that the capture regions total approximately 38 Mb. This kit covers the 1.22% of the human genome corresponding to the Consensus Conserved Domain Sequences database (CCDS) and greater than 1000 non-coding RNAs. Flowcell preparation and 76bp paired end read sequencing were carried out as per protocol for the GAIIx sequencer (Illumina Inc, San Diego CA)[27]. Approximately two lanes on a GAIIx flowcell were used per exome sample to generate sufficient reads to generate the aligned sequence.

Image analysis and base calling on all lanes of data were performed using Illumina Genome Analyzer Pipeline software (GAPipeline versions 1.4.0 or greater) with default parameters. The complexity of sequencing libraries was between 97.5%–99.3%. Complexity is the percentage of unique molecules determined from the first 25 bp in single-fragment reads and the combination of the first 25 bp in both paired-end reads out of total number of reads where the match chromosome field is not “QC” and the first 25 bp have no ‘N’s.

### Read mapping, variant calling, and annotation

Reads were aligned to a human reference sequence (UCSC assembly hg18, NCBI build 36) using the package called “efficient large-scale alignment of nucleotide databases” (ELAND). Reads that aligned uniquely were grouped into genomic sequence intervals of about 100 kb, and reads that failed to align were binned with their paired-end mates. Reads in each bin were subjected to a Smith-Waterman-based local alignment algorithm, *cross_match* using the parameters–minscore 21 and –mask level 0 to their respective 100kb genomic sequence (http://www.phrap.org). A total of 3.04–3.53 Gb of high-confidence mappable sequence data were generated in autosomal targeted regions per individual (Table S1). Genotypes were called at all positions where there were high-quality sequence bases (Phred-like Q20 or greater) using a Bayesian algorithm (Most Probable Genotype – MPG; Nancy F Hansen, unpublished observations). The genotype calls were compared against Illumina Human 1M-Quad genotype chips. Genotypes with a MPG score of 10 or greater show >99.89% concordance with SNP Chip data. The targeted regions included the exons of 17,134 genes and total 36,025,890 bases in the human genome. We successfully sequenced 92%–94% of targeted regions to an average depth per individual of 84–98 fold redundancy on the autosomes, 90%–94% of targeted regions and 57-93 fold redundancy on the X-chromosomes and 66%–68% of targeted regions and 45-51 fold redundancy on the Y chromosome in males. Of the total targeted sequence, 24–25 Mb of the genome corresponded to protein coding exons as per UCSC known gene annotation (Table S2). This resulted in capturing and sequencing of 79%–81% of the exome as defined by UCSC known gene annotations. Our custom protein variation annotation pipeline annotated an average of 8,585 missense and 87 termination changes per individual. Our annotation of cSNVs (coding single nucleotide variants) was based on UCSC all known genes. A number of filtering and prioritization steps were applied to reduce the number and to identify potentially pathogenic mutations, similar to the methods used in previous studies [28], [29]. Missense variants were sorted by the degree of severity of functional disruption prediction using CDPred. Variants detected in dbSNP (version 130) and in eight HapMap exome data were excluded from being potential disease causing cSNVs [29]. Imposing the requirement to satisfy all of the above three filters for severity of amino acid substitution, and not being a common variant resulted in 483-636 potential protein affecting variants (Table S2). Based on the inheritance pattern of the disease we required the variants to follow a Mendelian autosomal recessive pattern of inheritance, the homozygous subset of which yielded two potential genes (*AFG3L2, DMGDH*) (Table S3).

### Site-directed mutagenesis and yeast complementation

Wild-type or mutant variants of mature human AFG3L2 and human SPG7 were fused to the mitochondrial targeting sequence of the yeast *m*-AAA protease subunit Yta10 [17]. The human *AFG3L2* gene was mutagenized in yeast expression constructs using the QuikChange XL Site-Directed Mutagenesis Kit (Agilent). Mutations were verified by DNA sequencing. Mutant variants were co-expressed from the multicopy vector YEplac111 under the control of the *ADH1* promoter in Δ*yta10*Δ*yta12* yeast cells (YKO200)[30]. Yeast cells were grown under standard growth conditions at 30°C either in YP medium [1% (w/v) yeast extract, 2% (w/v) peptone] or minimal medium [0.67% yeast nitrogen base, 0.15% amino acid mix] supplemented with 40 µg/ml of adenine and tryptophan, 20 µg/ml of histidine and uracile, 60 µg/ml of leucine, and 30 µg/ml lysine in different combinations, both containing 2% (w/v) glucose or for isolation of mitochondria 2% (w/v) galactose and 0.5% (w/v) lactate. To test for respiratory chain activity, yeast cells were grown on YP medium containing 3% (w/v) glycerol as the sole carbon source. Expression of the human *m*-AAA protease subunits and processing of MrpL32 was assessed by immunoblotting using *m*-AAA protease and MrpL32-specific antibodies.

### Yeast mitochondrial isolation and BN-PAGE

The following procedures were performed as described previously: Yeast mitochondria isolation [31]; yeast BN-PAGE analysis [32]. BN-PAGE on human fibroblasts was performed as previously described using digitonin as detergent (detergent to protein ratio was 2∶1 [w/w]) [33].

### Cell transfection

(Figure S1) The coding region of murine *Afg3l2* (GenBank/EMBL/DDBJ accession no. NM_027130) was cloned into the SfiI-EcoRV sites of the vector pcDNA3.1 in frame with HA. The Y615C mutation (corresponding to the human Y616C) was introduced by site-directed mutagenesis.

To assess targeting of Afg3l2-HA to mitochondria, HeLa cells were co-transfected with a mitochondrially-targeted variant of GFP (Su9-GFP) and Afg3l2-WT or Afg3l2-Y615C constructs using Lipofectamine 2000 (Invitrogen). After 48 hours, cells were collected, fixed with PFA4%/PBS and immunofluorescence was performed as previously described (Errico et al. 2000). AFG3L2 was detected using specific antibodies (1∶200). Immunofluorescences were examined and images acquired using an Axioplan microscope equipped with an Apotome system (Zeiss).

## Supporting Information

Figure S1 Immunofluorescence analysis of HeLa cells co-transfected with wild-type murine *Afg3l2* or the mutant variant Y615C and a mitochondrially-targeted GFP construct (Su9-GFP). Both wild-type and mutant AFG3L2 proteins localize to mitochondria after overexpression.
(TIF)

Table S1 Sequencing details of targeted exomes of four individuals in a single family.
(DOC)

Table S2 Statistics of number of protein changing variants detected and various filters applied to not consider common and benign protein coding variation. * NS : Non-synonymous. ** Genotype calls with MPG score ≥10; (% of Exome (30,716,913); UCSC). ^‡^ HapMap8 : Coding variation from 8 HapMap Exomes^25^. ^‡‡^Damaging prediction by CDPred (very relaxed threshold).
(DOC)

Table S3 Number of genes detected with the application of homozygous recessive inheritance model.
(DOC)
