# Correction: Whole-Exome Sequencing Identifies Homozygous *AFG3L2* Mutations in a Spastic Ataxia-Neuropathy Syndrome Linked to Mitochondrial *m*-AAA Proteases

**DOI:** 10.1371/annotation/273d7d98-3a1b-494b-839e-de31a0f33d28

**Published:** 2013-02-27

**Authors:** Tyler Mark Pierson, David Adams, Florian Bonn, Paola Martinelli, Praveen F. Cherukuri, Jamie K. Teer, Nancy F. Hansen, Pedro Cruz, James C. Mullikin, Robert W. Blakesley, Gretchen Golas, Justin Kwan, Anthony Sandler, Karin Fuentes Fajardo, Thomas Markello, Cynthia Tifft, Craig Blackstone, Elena I. Rugarli, Thomas Langer, William A. Gahl, Camilo Toro

The XML annotation for the name of the 9th author, James C. Mullikin, is incorrect. The correct annotation should indicate that this author's surname is "Mullikin" rather than "Mullikin for the NISC Comparative Sequencing Program".

