# Supplementary material for: Whole-Exome Sequencing Identifies Homozygous AFG3L2 Mutations in a Spastic Ataxia-Neuropathy Syndrome Linked to Mitochondrial m-AAA Proteases
Source: PLoS Genet. 2011 Oct 13;7(10):e1002325. doi: 10.1371/journal.pgen.1002325 (PMC3192828; doi:10.1371/journal.pgen.1002325)
Supplement: Table S1 — Sequencing details of targeted exomes of four individuals in a single family. (DOC) [file pgen.1002325.s002.doc]

**Table S1.**

| **Sequencing Details** | | **IV.1** | **IV.2** | **III.1** | **III.2** |
| --- | --- | --- | --- | --- | --- |
| **Autosomes** | **Sequenced** | 33,225,255 | 33,114,311 | 32,988,471 | 32,578,809 |
|  | **Targeted** | 36,025,890 | 36,025,890 | 36,025,890 | 36,025,890 |
|  | **%TargetSeq** | 92% | 92% | 92% | 90% |
|  | **TotalBasesSeq** | 3.27E+09 | 3.53E+09 | 3.27E+09 | 3.04E+09 |
|  | **AvgSeqDepth** | 91X | 98X | 91X | 84X |
| **X-Chromosome** | **Sequenced** | 1,448,644 | 1,447,679 | 1,447,325 | 1,389,560 |
|  | **Targeted** | 1,537,242 | 1,537,242 | 1,537,242 | 1,537,242 |
|  | **%TargetSeq** | 94% | 94% | 94% | 90% |
|  | **TotalBasesSeq** | 8.82E+07 | 9.89E+07 | 8.93E+07 | 1.43E+08 |
|  | **AvgSeqDepth** | 57X | 64X | 58X | 93X |
| **Y-Chromosome** | **Sequenced** | 52,120 | 52,352 | 50,990 | 0 |
|  | **Targeted** | 77,264 | 77,264 | 77,264 | 77,264 |
|  | **%TargetSeq** | 67% | 68% | 66% | 0% |
|  | **TotalBasesSeq** | 3.45E+06 | 3.92E+06 | 3.50E+06 | 0.00E+00 |
|  | **AvgSeqDepth** | 45X | 51X | 45X | 0X |
