# Supplementary material for: Whole-Exome Sequencing Identifies Homozygous AFG3L2 Mutations in a Spastic Ataxia-Neuropathy Syndrome Linked to Mitochondrial m-AAA Proteases
Source: PLoS Genet. 2011 Oct 13;7(10):e1002325. doi: 10.1371/journal.pgen.1002325 (PMC3192828; doi:10.1371/journal.pgen.1002325)
Supplement: Table S2 — Statistics of number of protein changing variants detected and various filters applied to not consider common and benign protein coding variation. * NS : Non-synonymous. ** Genotype calls with MPG score ≥10; (% of Exome (30,716,913); UCSC). ‡ HapMap8 : Coding variation from 8 HapMap Exomes25. ‡‡Damaging prediction by CDPred (very relaxed threshold). (DOC) [file pgen.1002325.s003.doc]

**Table S2.**

| **Sample** | **Total**  **(NS* + Stop)** | **Not in dbSNP**  **(NS* + Stop)** | **Not in HapMap8^‡^**  **(NS* + Stop)** | **Not in Either**  **(NS* + Stop)** | **Not in Both AND Predicted damaging^‡‡^**  **(NS* + Stop)** | **Total Protein Coding Exon Bases Sequenced** (%)** |
| --- | --- | --- | --- | --- | --- | --- |
| IV.1 | 8433 + 86 | 1158 + 32 | 2280 + 47 | 954 + 27 | 567 + 27 | 24.78M (80.7%) |
| IV.2 | 8682 + 90 | 983 + 32 | 1839 + 42 | 800 + 27 | 456 + 27 | 24.66M (80.3%) |
| III.1 | 8680 + 93 | 1236 + 37 | 2349 + 53 | 1014 + 33 | 603 + 33 | 24.62M (80.1%) |
| III.2 | 8546 + 79 | 1099 + 31 | 2339 + 50 | 945 + 30 | 549 + 30 | 24.25M (78.9%) |
