# Supplementary material for: Whole-Exome Sequencing Identifies Homozygous AFG3L2 Mutations in a Spastic Ataxia-Neuropathy Syndrome Linked to Mitochondrial m-AAA Proteases
Source: PLoS Genet. 2011 Oct 13;7(10):e1002325. doi: 10.1371/journal.pgen.1002325 (PMC3192828; doi:10.1371/journal.pgen.1002325)
Supplement: Table S3 — Number of genes detected with the application of homozygous recessive inheritance model. (DOC) [file pgen.1002325.s004.doc]

**Table S3.**

| **Inheritance Model** | **NS, Stop, Splice** | **Not in dbSNP** | | **Not in dbSNP AND Damaging** | |
| --- | --- | --- | --- | --- | --- |
|  | **Number of Genes** | **Number of Genes** | **Gene Short Names** | **Number of Genes** | **Gene Short Names** |
| **Homozygous**  **Recessive Inheritance Model** | 71 | 7 | *AFG3L2*  *BTNL8*  *CDC27 DMGDH SLC5A9 ZNF717*  *ZNFX1* | 2 | ***AFG3L2***  *DMGDH* |
